# Life‐long cardiorespiratory consequences of early life stress on estrogen actions in female rats

**DOI:** 10.1111/jne.70309

**Published:** 2026-11-05

**Authors:** Richard Kinkead, Stéphanie Fournier, Luana Tenorio‐Lopes, Danuzia Ambrozio‐Marques

**Affiliations:** ^1^ Department of Pediatrics, Faculty of Medicine Université Laval Québec Québec Canada; ^2^ Research Center of the Québec Heart & Lung Institute Université Laval Québec Québec Canada; ^3^ Department of Physiology, Institute of Biosciences UNESP Botucatu Botucatu Brazil

**Keywords:** ageing, hypertension, neonatal maternal separation, panic disorder, sleep apnea

## Abstract

Early‐life adversity increases risk for disease with sex‐specific effects on brain development. While the association between early‐life stress and neuropsychiatric disorders is well established, its impact on respiratory health is poorly documented, particularly in females. Here, we review recent studies using neonatal maternal separation (NMS) to examine the long‐term consequences of early‐life stress on cardiorespiratory function in females. Panic disorder (PD) is an anxiety disorder that is ⁓2 times more prevalent in women. The respiratory subtype is characterised by an exaggerated ventilatory response to CO_2_. NMS augmented the response to 5% CO_2_ of adult female rats; this effect was most pronounced during proestrus and absent in males. Orexin (ORX) neurons potentiate the CO_2_ response and are normally inhibited by 17β‐estradiol (E2). Electrophysiological measurements showed that in control females, excitatory postsynaptic current frequency varied inversely with E2 levels. However, NMS disrupted this E2‐mediated inhibition, particularly during proestrus, implicating impaired estrogen action in PD‐related respiratory dysfunction. Age‐related ovarian decline increases the risk of sleep apnea and associated comorbidities. Whereas apnea frequency decreased with age in control females, 1 year‐old NMS females exhibited a 75% higher apnea index than age‐matched controls, together with greater adiposity and hypertension. Arterial pressure was inversely correlated with circulating E2 levels only in NMS females, indicating heightened vulnerability to age‐related estrogen decline. Although physiological fluctuations in E2 levels exert modest effects in healthy females, we propose that early‐life stress persistently alters responsiveness to E2 and acts synergistically with ageing to increase cardiovascular vulnerability throughout the female lifespan.

## 1 INTRODUCTION

Forty years ago, Barker and Osmond published their seminal epidemiological study on the origins of ischemic heart disease in England and Wales. Their investigation led them to conclude that “Ischaemic heart disease is strongly correlated with both neonatal and post‐neonatal mortality and to suggest that poor nutrition in early life increases susceptibility to the effects of an affluent diet”.^1^ This work was impactful at the time as its conclusion shifted scientific focus from genetics and adult behaviours to early developmental environments as determinants of lifelong health. This study has stood the test of time and has shed the basis for a new research perspective known today as the “Developmental Origins of Health and Disease” (DOHaD). Accordingly, a growing scientific consensus suggests that many adult diseases originate from developmental or biological disruptions occurring early in life. These early‐life experiences can influence adult health in two main ways: through the cumulative buildup of damage over time, or through the biological embedding of adversity during sensitive periods of development. In both situations, there may be a delay of many years or decades before these early adverse experiences manifest as disease.^2^ Accordingly, enthusiastic supporters of this view have proposed an interesting (and debatable) view that “*from both research and policy perspectives, addressing the roots of physical and mental health disparities early in life is likely to be far more effective than attempting to change health‐related behaviours or expanding access to healthcare only in adulthood*”.^2^

Regardless, today, many biomedical disciplines acknowledge early life stress as an important risk for disease and consider stress‐related neuroendocrine disturbances in pathophysiological investigations. But by far, the association between stress and mental health issues is more accepted and considered in psychology and psychiatry than in studies of “hard core” physiological systems. Sleep apnea (SA) and asthma are arguably the two most important respiratory disorders, and while stress‐related mental health issues such as anxiety and depression nearly double the risk of these diseases,^3^, ^4^ the link between stress and these important health issues has received limited attention. Part of the problem is that addressing this link in humans is difficult, as stress comes in many forms and is often associated with numerous lifestyle‐related confounding factors.

This gap in knowledge combined with a need to address respiratory disorders from a different angle has led us to propose that early life stress increases vulnerability to respiratory disorders by disrupting development and function of the neural networks that control breathing. Over the years, we tested this hypothesis by subjecting rats to various stress models and the results demonstrate that exposure to perinatal stress results in respiratory disturbances that are generally in line with clinical conditions such as apnea of prematurity, panic attacks, and SA; the deleterious consequences of perinatal stress on the respiratory system not only persist well into adulthood but also show significant sex‐based differences that are consistent with clinical observations. Readers are referred to two extensive reviews for further details.^5^, ^6^ This is important because although manifestations of those important respiratory disorders vary between men and women, the origins of those sex‐based differences are poorly understood, as women are underrepresented in clinical research and until recently, females were rarely used in preclinical work. Having said this, the sex‐specific effects of early‐life stress on physiological outcomes in rats offer a unique opportunity to address this gap in knowledge and sex‐based inequity in respiratory research. With that in mind, the main goal of this review is to discuss recent advances from our laboratory documenting how neonatal maternal separation (NMS), an established model of early‐life stress, leads to cardiorespiratory and metabolic disturbances across various life stages and explore the contribution of estrogen's actions to that problem.

## 2 NEONATAL MATERNAL SEPARATION AS A MODEL OF EARLY LIFE STRESS

In mammals (including humans), the tactile, olfactory, and auditory stimuli that the mother provides to her offspring are amongst the most potent external signals affecting brain development of the newborn.^7^, ^8^ Consequently, adverse conditions such as unstable/inadequate parental environment and specialised medical care that interferes with mother‐infant interactions compromise neurological outcomes.^2^, ^7^, ^9^, ^10^, ^11^ Abnormal/insufficient interactions between the mother and her offspring disrupt the balance between excitatory and inhibitory signals regulating the paraventricular nucleus of the hypothalamus (PVH), a structure that orchestrates the release of stress peptides and hormones.^12^ Inadequate maternal care therefore augments basal activity of the hypothalamo‐pituitary–adrenal (HPA) axis and potentiates the responsiveness to stress throughout life.^13^, ^14^ Much like chronic stress, this condition augments the risk for a broad range of health issues, including depression, anxiety, diabetes, and cardiovascular diseases,^2^, ^15^ which are significant co‐morbidities of respiratory disorders such as SA.^16^

From a “respiratory perspective,” it is important to highlight that the PVH sends direct projections onto medullary structures that (1) integrate chemical and mechanical stimuli, (2) generate the respiratory rhythm, and (3) activate the main respiratory muscles including the diaphragm and upper airways (Figure 1). Our previous work showing that in males, NMS‐induced PVH dysfunction augments the ventilatory response to hypoxia and augments respiratory instability during sleep illustrates this concept (reviewed in^5^). From an experimental perspective, disrupting mother‐pup interactions offers a simple, robust, and persistent means of disturbing the HPA axis and exploring its impacts on respiratory phenotypes. Furthermore, this animal model offers better control over confounding environmental and lifestyle factors that often limit the conclusions drawn from clinical studies on stress.

**FIGURE 1:**
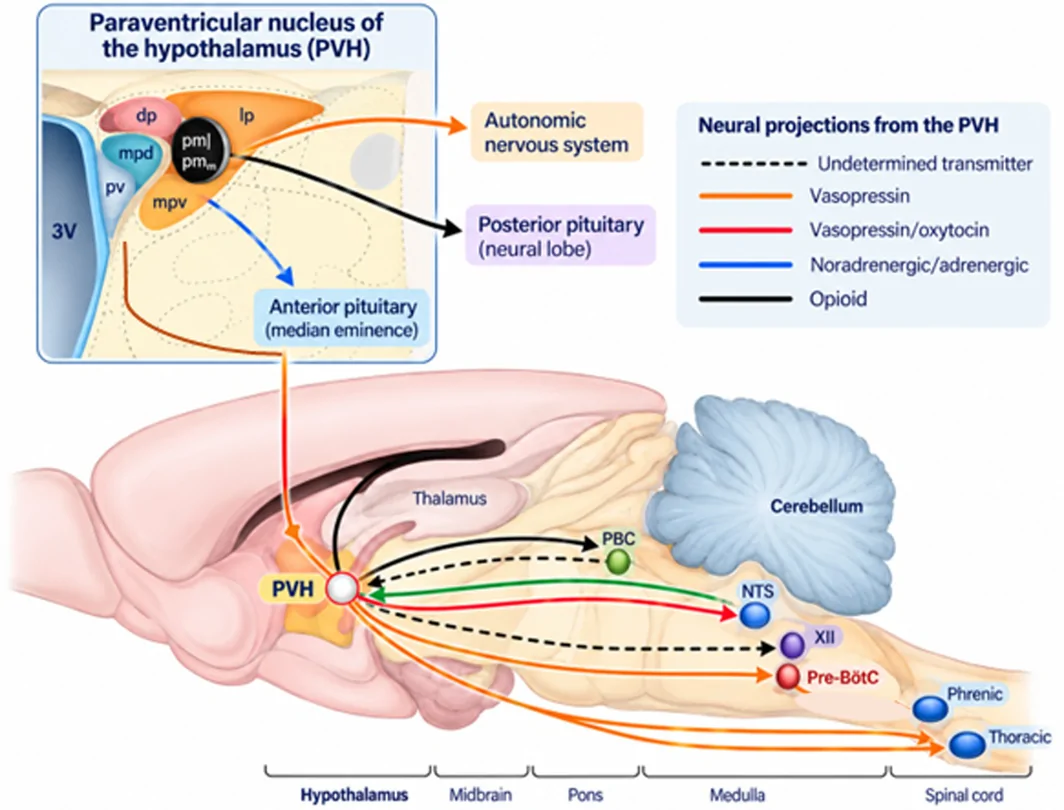
Neuroanatomy of the paraventricular nucleus of the hypothalamus (PVH) has numerous anatomical projections onto key structures of the respiratory control system. Top panel: Coronal view of the PVH illustrating its main subdivisions and general projections. *Abbreviations*: dp, dorsal parvocellular part; lp, lateral parvocellular part; mpd, pv, dorsal and ventral subdivisions of the medial parvocellular part (CRH); pml, pmm, lateral (vasopressinergic) and medial (oxytocinergic) subdivisions of the posterior magnocellular part; mpv, periventricular part. Lower panel: Sagittal view of the rat brain from^17^ showing projections from the paraventricular nucleus to key groups of neurons involved in the genesis and regulation of respiratory activity. The colours indicate the predominant transmitters identified. Note that the interaction between the PVH and some nuclei is bidirectional. PBC, parabrachial complex; NTS, nucleus of the solitary tract; XII, hypoglossal motor nucleus; Pre‐Böt C, Pre‐Bötzinger complex. Created with Biorender.com. Information based on Swanson LW and Sawchenko PE, 1983,^18^; Adapted from Swanson LW, 2018^17^; the original figure was adapted, with permission, from^5^ and visually redesigned using ChatGPT (OpenAI). The scientific content was reviewed by the authors.

In addition to environmental factors, gonadal hormones are another key determinant of HPA axis development and function and in rodents and humans, sex‐based differences in the stress response emerge shortly after birth.^19^ Since the gonado‐ and corticotropic axes are both anatomically and functionally inter‐related,^20^ early life stress interferes with their sexual differentiation.^21^, ^22^ Consistent with this basic principle, several laboratories (including ours) have established the sex‐specific effects of early‐life stress on the HPA axis.^14^, ^23^ Using NMS as a stress model, we demonstrated that at sexual maturity (8 weeks), basal activation of the PVH and plasma levels of corticotropin (ACTH) and corticosterone of stressed male rats are greater than controls; however, this was not observed in NMS females.^14^ Neonatal maternal separation therefore offers a unique model to investigate how sex steroids contribute to sex‐based differences in respiratory disorders.

## 3 OBJECTIVES AND OVERVIEW

Guided by this opportunity, this review discusses the lifelong cardiorespiratory consequences of early life stress in female rats. We revisit recent evidence demonstrating that anomalies in the responses to estrogens are an important pathophysiological mechanism. We compare the respiratory function of NMS and control females at key stages of reproductive function (sexual maturity, “middle‐age”, and ovarian senescence). As respiratory issues are often accompanied by significant co‐morbidities, we also compare cardiovascular and metabolic indices of NMS and control females; the time‐line of the project is presented schematically on Figure 2.

**FIGURE 2:**
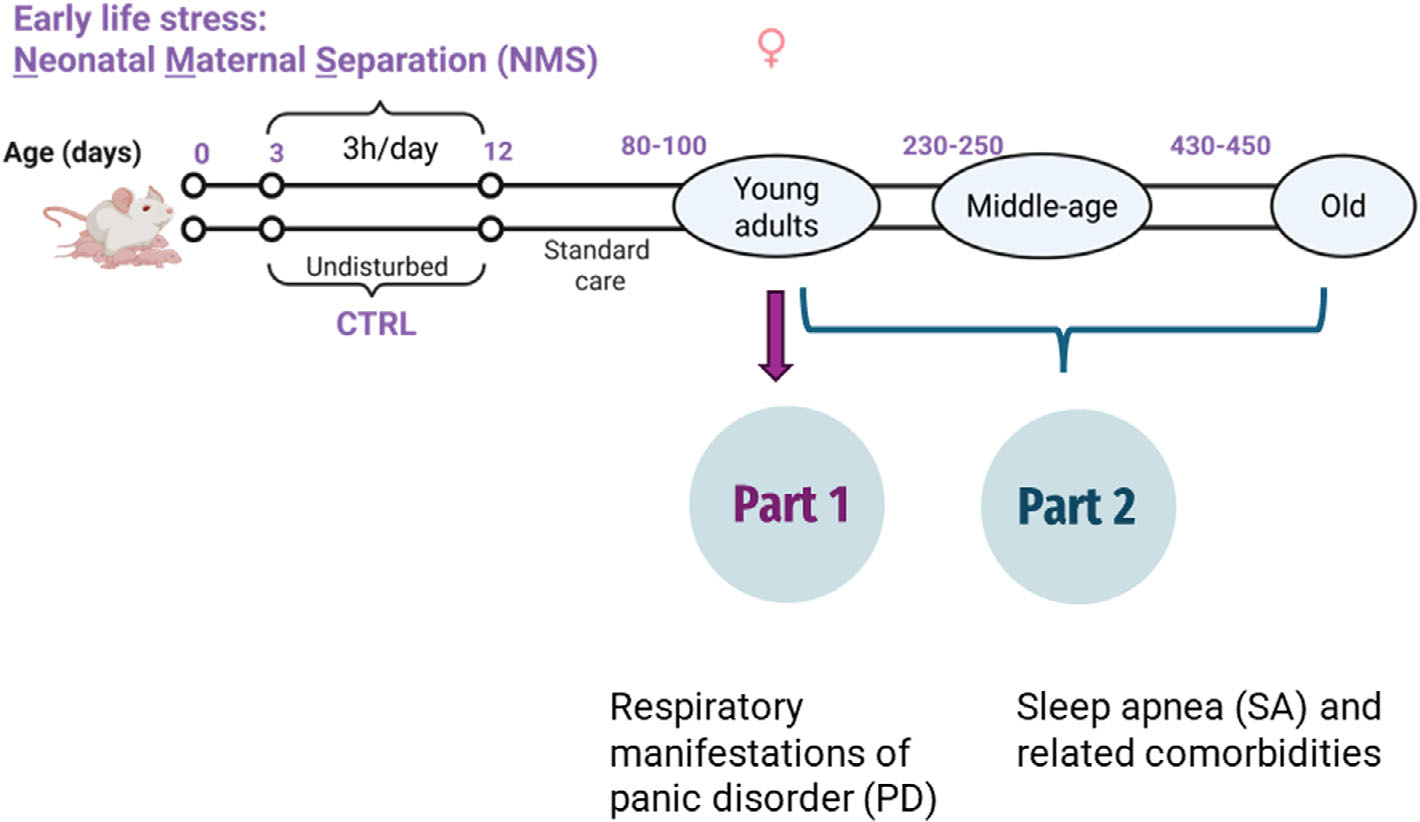
Timeline of the study design highlighting the experimental treatments and the three age groups and respiratory disorders discussed in the review. Created with BioRender; adapted from^24^ under the terms of the Creative Commons Attribution License. © 2026.

Panic disorder (PD) is an anxiety disorder which is ⁓2 times more prevalent in women than men.^25^ Different subtypes of PD have been described, including cardiac, vestibular, and respiratory subtypes.^26^, ^27^ Because an excessive ventilatory response to CO_2_ is a hallmark of the respiratory subtype of PD, the first section discusses how NMS leads to this abnormal phenotype in young adults. The second part summarises recent results showing how NMS alters the ageing trajectory of females and favours the emergence of a cardiorespiratory and metabolic phenotype similar to that of women who develop SA at menopause (Figure 2). While preclinical research offers important insights into the underlying pathophysiology of disease, caution is warranted when extrapolating these findings to humans, as reproductive senescence in rodents differs markedly from human menopause. By comparison with humans, rat senescence is more heterogeneous, with central neuroendocrine failure, disrupted hormone surges, and variable residual production of ovarian steroid, including E2.^28^

## 4 MAIN EXPERIMENTAL APPROACHES

### 4.1 Neonatal maternal separation

All experiments were performed on animals born and raised in our animal care facilities (Université Laval). Following birth, the entire litter is separated daily from their mother for 3 h/day from postnatal days 3 to 12. Pups were placed in an incubator with controlled humidity (45%) and temperature (35°C) to maintain thermoneutrality.^29^ This protocol reduces mother‐pup interactions beyond the normal intervals between nursing bouts with minimal effects on food intake and growth. On day 21, rats were weaned and reared under standard conditions until they were used for experiments at sexual maturity (8 weeks old or later^30^). Data from NMS rats are compared to those that were not disturbed during the first 2 weeks of life; this is the most desirable control group for investigating the effects of NMS.^11^ Each group is composed of animals from at least three different mothers to avoid litter‐specific effects.

### 4.2 Respiratory measurements with whole body plethysmography

This is the most reliable approach for non‐invasive measurement of respiratory variables (breathing frequency, tidal volume, minute ventilation) in unrestrained animals.^31^ It also allows detection of sighs and apneas. Rats are acclimated to the plethysmography chamber (⁓1 h) and recording of basal respiratory parameters (frequency, tidal volume, and minute ventilation) then begins when the animal appears calm and the respiratory signal is exempt of artefacts due to movement or sniffing. Modification of the inflowing gas mixture allows measurement of the ventilatory responses to respiratory stimuli such as increased CO_2_ (hypercapnia) or reduced O_2_ (hypoxia). A representative recording is presented in Figure 3A.

**FIGURE 3:**
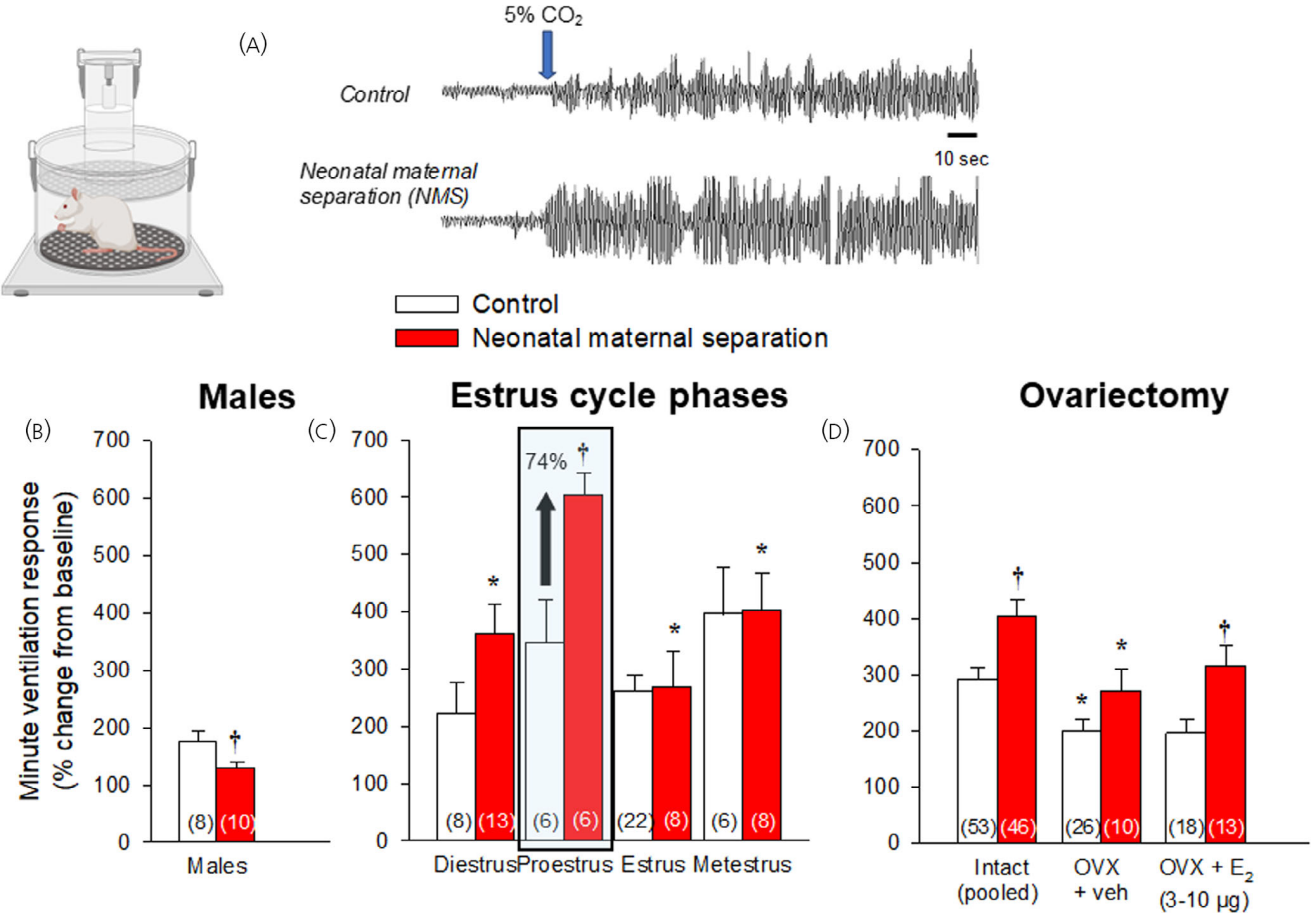
(A) Illustration of the recording chamber and original plethysmographic signals comparing ventilatory activity at rest and at the onset of CO_2_ inhalation (5% CO_2_; blue arrow) in a female raised under control conditions (top trace) versus a female subjected to neonatal maternal separation (bottom trace; NMS: 3 h/day; postnatal days 3–12). Comparison of the mean minute ventilation response to CO_2_ inhalation (5% CO_2_; 10 min) between (B) adult male and (C) female raised under standard conditions or subjected to neonatal maternal separation (NMS) across the different phase of the estrus cycle. Female data also consider the estrus cycle phases; the blue area emphasises the proestrus phase, when the group differences were greatest. (D) effect of ovariectomy and 17β‐estradiol (E2) supplementation on the mean minute ventilation response to CO_2_ of females subjected to NMS or maintained under standard conditions. Note that for the intact group, results from various estrus cycle phases were pooled. E2 replacement reproduced cyclic fluctuations by performing a daily injection every 4 days over 12 days prior to the experiments (4 injections in total). Each injection contained either vehicle (peanut oil, 100 μL) or E2 (3 or 10 μg); these doses restore normal E2 levels. The minute ventilation response is expressed as a percentage change from baseline (room air). Data are reported as means ± SEM. The numbers in brackets below the boxes indicate the number of replicates in each group. Post hoc pairwise comparisons were performed only when warranted by ANOVA. *Indicates a value different from corresponding proestrus value at *p* < 0.05. **†**Indicates a value different from corresponding control value at *p* < 0.05. Adapted, with permission from.^32^

## 5 PART I: RESPIRATORY MANIFESTATIONS OF PANIC DISORDER (PD): EARLY LIFE STRESS AND DISRUPTION OF ESTRADIOL REGULATION OF OREXIN NEURONS

### 5.1 Panic disorder: Definition, etiology, and sex‐based differences

Panic disorder (PD) is an anxiety disorder characterised by recurrent panic attacks that are acute and unexpected.^33^ A panic attack is defined as an episode of overwhelming physical distress and cognitive anxiety during which the patient rapidly develops intense symptoms including air hunger, sweating, heart palpitations, shortness of breath, hyperventilation, and fear of dying.^34^ As such, PD is perhaps one of the most overwhelming experiences that a person can endure.^35^ Historically, PD was first reported during World War I when it was observed that some soldiers were unable to keep their gas mask in place despite being exposed to life‐threatening conditions.^36^ It was then discovered that these soldiers were excessively sensitive to the difficulty of breathing with this mask and the impoverished air quality/CO_2_ build up in their apparatus. Today, the DSM‐V definition of PD spans across 13 different symptoms; a respiratory PD subtype has been identified as the most common, the most pervasive, and the most disabling form of PD.^37^ Respiratory distress being a prominent symptom of PD, abnormalities in the perception of, and the excessive responses to, respiratory stimuli are hence central to the pathophysiology of this condition.^38^, ^39^ CO_2_ inhalation is commonly used as a diagnostic tool for PD, and an increased CO_2_ response is acknowledged as a distinctive biomarker of this population.^34^, ^40^ In preclinical research, evaluation of the cardiorespiratory and behavioural responses to CO_2_ inhalation are useful metrics to study the neurobiology of anxiety and panic states.^41^, ^42^, ^43^, ^44^

In North America, PD affects ~5% of the population.^34^ What is striking, however, is that in women, the prevalence of PD or an enhanced response to CO_2_ inhalation is 2 to 3 times that of men.^45^ Gene‐by‐environment interactions contribute to liability for PD and its developmental precursor: separation anxiety disorder. Childhood adversity, particularly parental loss, is associated with increased risk for panic disorder and CO_2_ hypersensitivity.^41^

The origins and neuroendocrine underpinnings for the sex‐based difference in respiratory dysfunction observed in PD patients remain poorly understood^45^; however, the rise of PD prevalence at puberty and the fact that the panicogenic effects of CO_2_ inhalation peak during the pre‐menstrual phase indicate that women suffering from PD are more sensitive to hormonal fluctuations than healthy subjects.^25^, ^46^ Cyclic fluctuations in ovarian hormones are normal physiological processes, but in a subpopulation of women, these events affect the onset and recurrent exacerbations of PD.^47^ Thus, elucidating why hormonal fluctuations lead to respiratory manifestations of PD is a key question that has been driving our research.

### 5.2 Neonatal maternal separation: A relevant model to investigate PD

As the ventilatory response to CO_2_ is an evolutionarily conserved trait, its quantification has enabled translational studies in animal models of early parental separation. Consistent with human findings, experimental maternal separation in rodents increases CO_2_ sensitivity, with evidence in mice supporting gene‐by‐environment interaction effects.^48^ Despite acknowledged species differences, parallel molecular genetic investigations in longitudinal human cohorts and controlled animal models provide a valuable framework for elucidating underlying mechanisms.^9^ Using rat as a model system, our laboratory has consistently demonstrated that NMS is sufficient to disrupt development of the neural circuits that regulate breathing in a persistent and sex‐specific fashion.^5^ At adulthood, the respiratory phenotype of rats subjected to NMS displays several features reported in PD patients, including a sex‐specific enhancement of the hyperventilatory response to a CO_2_ enriched gas mixture (5% CO_2_ in air); this CO_2_ level is based on protocols used in the clinic for CO_2_ inhalation tests.^49^ Specifically, the response of NMS females is significantly larger than controls; this was not observed in males (Figure 3B). Furthermore, NMS‐related enhancement of the CO_2_ response becomes apparent only after puberty and in adults, it fluctuates with the estrus cycle, peaking during proestrus; at this stage, the response of NMS females is 74% greater than controls (Figure 3C). Ovariectomy (OVX) reduces the CO_2_ response of NMS and control females similarly but the fact that subsequent 17β‐estradiol (E2) replacement has no effect in controls but restores the CO_2_ response of NMS females near pre‐surgical levels indicates that these females are more sensitive to changes in E2; that this hormone may therefore be a main determinant of CO_2_ hypersensitivity (Figure 3D).

### 5.3 Stress‐related dysfunction of orexin (ORX) neurons: A core mechanism in sex‐based differences in CO_2_ hyper‐responsiveness

Orexin A and B (also known as hypocretins) are regulatory peptides produced by neurons located in the lateral hypothalamus. Initially known for their role in the control of appetite, ORX neurons are now recognised for their powerful influence on the regulation of the sleep/wake cycles and the stress response.^50^, ^51^, ^52^, ^53^ Orexin neurons are divided according to their location within the hypothalamus: lateral hypothalamus (LH), perifornical area (PeF), and dorsomedial hypothalamus (DMH). These neurons have extensive projections throughout the central nervous system; however, the organisation of the ORX system is dichotomous: LH neurons stimulate motivated behaviours such as appetite for food and other rewards such as abused drugs,^54^ whereas neurons of the PeF and DMH act in parallel to influence arousal, sleep/wake states, and cardiorespiratory function.^55^, ^56^, ^57^, ^58^ Interestingly, the DMH/PeF region was initially termed the “panic area” because its activation induced a “panic‐like state” in experimental animals.^59^, ^60^

Orexin neurons project to parvocellular neurons of the PVH where they promote release of the corticotropin releasing factor (CRF), a key peptide in the neuroendocrine response to stress.^61^ Conversely, ORX neurons express CRF receptors^61^ and receive afferent signals from many stress‐related regions, including the PVH.^62^ This bidirectional relationship with stress‐related regions allows ORX neurons to play an essential role in the integration of stress signals. However, intense or prolonged stress exposure can initiate a positive feedback loop between the 2 systems^63^ which may explain why activation of orexin neurons is associated with anxiety and PD.^57^, ^63^

Orexin neurons also project to multiple structures generating and regulating breathing, which is why they are important determinants of respiratory activity also.^64^, ^65^, ^66^ Genetic deletion of ORX neurons or pharmacological inactivation of ORX receptors have limited effects on respiratory activity at rest but impair reflexive responses to ventilatory stimuli.^64^, ^65^, ^66^, ^67^ Because ORX synthesis is activity‐dependent, highly plastic, and responsive to stress,^68^ NMS augments basal ORX function and this stress‐related effect may potentiate the ventilatory response to CO_2_ inhalation.^32^, ^69^ Moreover, regulation of ORX function is sex‐specific.^70^ In males (rodents and humans), ORX levels decrease with ageing^71^, ^72^ whereas in women, plasma levels of ORX_A_ rise at menopause^73^, ^74^ due to the withdrawal of the E_2_‐related inhibition of ORX neurons and the stress pathways.^75^, ^76^, ^77^, ^78^ Results from preclinical experiments support the role of ORX neurons in CO_2_ hyperresponsiveness as pre‐treatment of females with a selective ORX‐1 receptor antagonist (SB334867; 15 mg/kg) attenuates the excessive ventilatory response to CO_2_ of NMS females but has no significant effect in controls. Of note, this attenuation is greatest when NMS females are in proestrus, a phase when E2 levels are highest.^32^

We then evaluated the effects of NMS on ORX neurons at the cellular level with an electrophysiological approach (patch clamp). Because ORX neurons are not organised in a dense, heterogenous structure, rats were injected with adeno‐associated virus (AAV) construct expressing green fluorescent protein (GFP) under the control of the ORX promoter (Figure 4A). Whole cell recording in voltage clamp configuration allows measurement of excitatory post‐synaptic currents (EPSCs) as the frequency and amplitude of those events reflect synaptic activity converging onto ORX neurons. Performing experiments at various stages of the estrus cycle and OVX females showed that, in line with E2‐related inhibition of ORX neurons,^75^, ^76^ the EPSC frequency of control rats was inversely related to the E2 levels measured in the plasma even though E2 levels did not differ between NMS and controls (Figure 4B,C). Conversely, this relationship was not observed in ORX neurons from NMS females and, unlike controls, the highest EPSC frequency was recorded during proestrus, the phase when the CO_2_ response is highest (and ORX antagonist is most effective) (Figure 4C).

**FIGURE 4:**
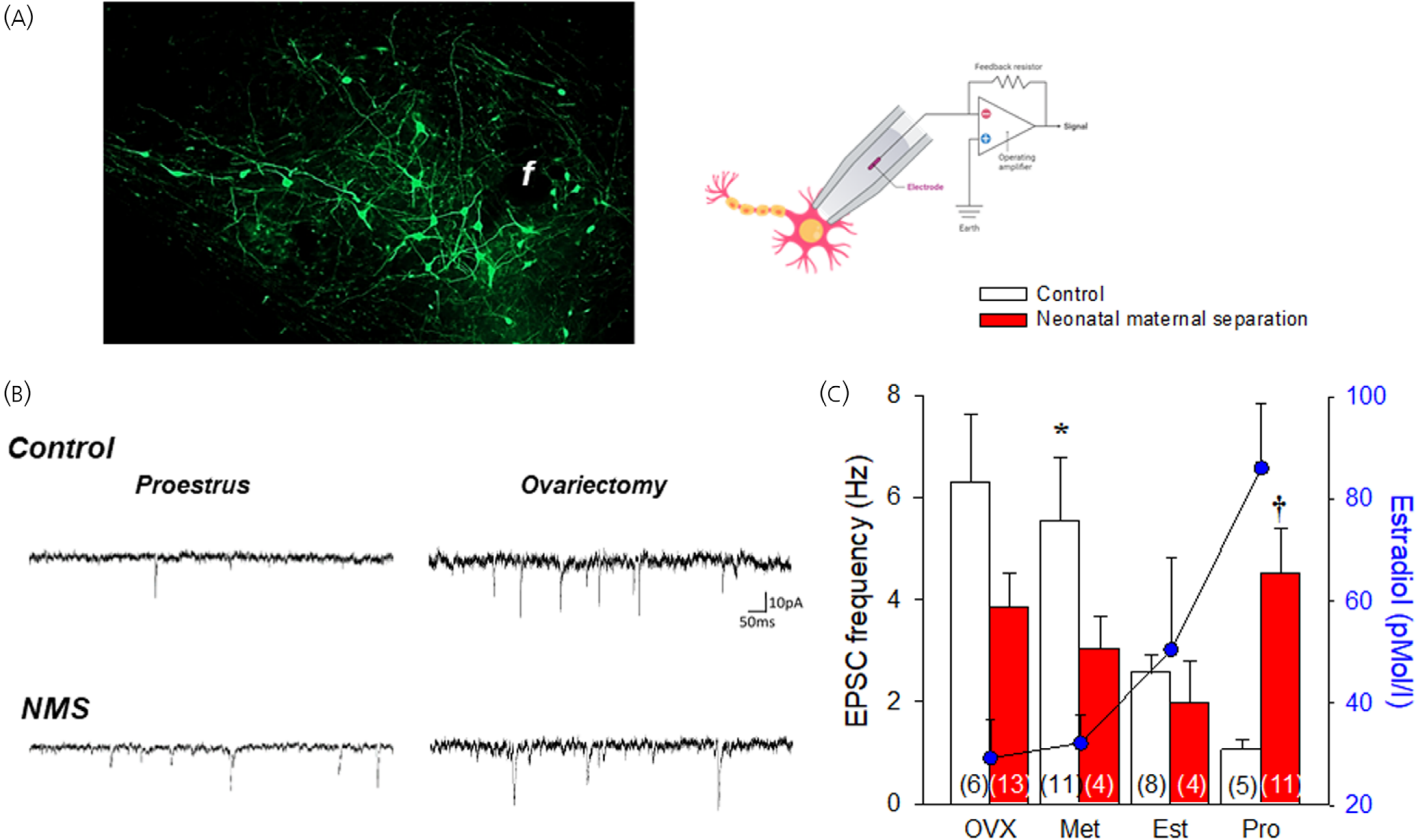
Neonatal maternal separation (NMS) reduces the excitatory postsynaptic currents (EPSC) recorded in GFP‐labeled orexin neurons in response to natural and ovariectomy‐induced changes in 17β‐estradiol (E2) level. (A) Photomicrograph illustrating GFP‐labeled orexin neurons; the fornix (f) is shown as a landmark. (B) Comparison of EPSC recordings from orexin neurons between cells obtained from females during proestrus and 2 weeks following ovariectomy (OVX); tissue slices originated from females raised under control conditions (top traces) or subjected to neonatal maternal separation (NMS, bottom traces; 3 h/day, postnatal days 3–12). (C) Population data of EPSC frequencies recorded during three distinct phases of the estrus cycle and following OVX. Data are expressed as the mean ± SEM. Post hoc pairwise comparisons were performed only when warranted by ANOVA. **†**Significantly different from corresponding control value at *p* ≤ 0.05. *Indicates a value statistically different from corresponding proestrus value at *p* < 0.05. Modified with permission from.^32^

In summary, these observations show that basal activity of ORX neurons of females previously subjected to NMS is higher than controls; an effect that contributes to excessive responsiveness to CO_2_ inhalation. The enhanced EPSC frequency reported in NMS females likely reflects greater excitatory drive converging onto ORX neurons, and while NMS compromises the inhibitory action of E2 on this signal, its origin remains unknown. As NMS does not alter HPA axis function, a contribution from the PVH is unlikely. However, astrocytes were recently shown to contribute to sex‐specific changes in ORX neuron firing in the LH of NMS‐subjected mice.^79^ In light of the evidence indicating that astrocytes contribute to central CO_2_ chemosensitivity, NMS‐related astrocyte remodelling may contribute to the excessive response of NMS females to CO_2_ inhalation.

### 5.4 Part I: Limitations and conclusion

Inadequate modelling of human disease hinders the translation of basic knowledge into effective treatments for humans.^80^ While our study shows that NMS closely reproduces the developmental and cyclic changes in the respiratory manifestations of PD, as well as enhanced ORX modulation, it is important to bear in mind that animal research cannot reproduce the complex psychosocial reality often associated with PD. Furthermore, the estrous cycle in rodents is not equivalent to the menstrual cycle in humans.^81^ Nevertheless, NMS meets key criteria expected of an animal model, including time‐dependent and sex‐specific effects on respiration.^35^, ^82^ The results reported here therefore provide valuable insights into the basic mechanisms underlying this neurological disorder in female rats. Our demonstration that NMS disrupts the inhibitory actions of E2 on respiratory control is significant, as it offers new avenues for alleviating PD.

## 6 PART II: EARLY LIFE STRESS ALTERS THE CARDIORESPIRATORY AGEING TRAJECTORY OF FEMALE RATS

### 6.1 Menopause: A turning point in the cardiorespiratory health of ageing women

The global population is ageing rapidly, with projections indicating that by 2050, approximately one‐fifth of the world's population will be aged 60 years or older, and women will constitute approximately 54% of this age group.^83^ Ageing increases the prevalence of chronic conditions and for women, the complex hormonal and physiological changes that take place during menopause can exacerbate age‐related risk. Sleep is a critical determinant of health and plays a fundamental role in maintaining brain and physiological functions across the lifespan.^84^ Advancing age is commonly associated with longer sleep latency, reduced sleep quality, and increased sleep fragility.^85^ Among age‐related sleep disorders, SA is a common yet frequently underdiagnosed condition, affecting nearly 1 billion adults worldwide.^86^ Sleep apnea is characterised by recurrent episodes of partial or complete interruption of airflow to the lungs during sleep; airway obstruction is the main cause of apneas, but regardless of its pathophysiological origins, SA leads to intermittent hypoxemia and repeated sleep fragmentation. These apneic events activate sympathetic and stress‐related pathways, promoting systemic inflammation,^87^ and are associated with excessive daytime sleepiness as well as long‐term health consequences. The prevalence and clinical manifestations of SA exhibit marked sex‐related differences,^6^ but because SA occurs approximately twice as frequently in men as in women, it has long been considered a “man's disease,” leading to frequent misdiagnosis of women reporting sleep‐related symptoms.^88^ This bias is problematic, as women with SA report sleep disturbances more often than men.^6^, ^89^ Nevertheless, it is now well established that the incidence of SA increases after menopause and reaches levels comparable to those observed in men.^90^

The pathophysiology of SA is complex and multifactorial; however, upper airway obstruction and instability of neural respiratory control are key contributing factors.^91^ Obesity further aggravates SA, as adipose tissue deposition in the neck region promotes airway collapse and hypoventilation, especially when sleeping in a supine position.^92^ Accordingly, body mass index (BMI) is positively correlated with SA severity and although BMI often increases during menopause, differences in SA severity between pre‐ and postmenopausal women persist after adjustment for BMI and neck circumference.^93^, ^94^ These findings suggest that, compared with men, the mechanisms underlying SA in postmenopausal women may rely more on abnormalities in respiratory control rather than on anatomical differences of the upper airway.^95^ However, this hypothesis remains to be tested experimentally.

Both ageing and SA are recognized risk factors for major health disorders, including hypertension, metabolic dysfunction, depression, and obesity.^96^, ^97^ Hypertension is of great interest as it represents a leading cause of global morbidity and mortality. Much like SA, hypertension has long been perceived as predominantly affecting men and shows a marked increase in prevalence among women after menopause, surpassing that of men around the age of 60.^98^, ^99^ The progressive loss of the “protective effects” of ovarian hormones is frequently invoked to explain the higher incidence of cardiovascular diseases observed after menopause.^6^, ^95^, ^100^ While menopause is a natural life transition, it remains unclear why, for a subpopulation of women, this process is associated with a greater risk of cardiorespiratory and metabolic disorders.

### 6.2 Does the impact of early life stress on health remain latent until ovarian function declines?

Stress exerts persistent and sex‐specific effects on the brain, and although altered neural control of breathing represents a key pathophysiological trait of SA, the contribution of stress to the age‐dependent emergence of this disorder in women has not yet been investigated. These considerations raise the possibility that, in women, the adverse effects of early‐life stress on respiratory health and related comorbidities remain latent until the onset of menopause. We tested this hypothesis experimentally by comparing the ageing trajectory of control female rats raised under standard housing conditions with that of females subjected to NMS. Specifically, we first measured the apnea index during sleep, respiratory reflexes, and cardiovascular function at three distinct ages: young adulthood (~8 weeks), middle age (~40 weeks), and old age (~60 weeks) (Figure 2). The potential contribution of obesity to age‐related changes in respiratory control was evaluated through assessments of body composition and leptin and cytokine signalling. Immunohistochemical analyses were conducted in selected brain regions to determine how NMS and ageing influence baseline neuronal activity and cellular structure within key components of the respiratory control network.

We monitored the hormonal profile across the three distinct age groups in both experimental groups and data show that in young females, E2 levels of NMS females were 32% higher than controls. Estradiol concentrations of control animals remained stable across ages but dropped progressively with age in NMS females such that by old age, E2 levels were 27% lower than in age‐matched controls. Despite suggestive trends, the age‐related decline in progesterone was not significant; NMS exposure did not affect progesterone levels. With regard to stress hormones, corticosterone levels were not affected by NMS or age. Conversely, ACTH concentrations augmented with age in both groups; however, values of old NMS were slightly lower than controls.^24^

Thus, at first, females seem protected against the sequelae of early‐life stress on HPA axis function.^14^ Age‐related drop in ovarian function increases responsiveness to acute stress^101^ and E_2_ contributes to this protection by preventing excessive activation of the stress pathways.^78^ Our data showing that in young adults, NMS augments HPA axis function in males but not females is in line with this concept. However, it is possible that the consequences of ageing on respiratory control during sleep are greater in women with a latent vulnerability due to previous adverse life experiences. We recently completed a series of experiments to address this issue in rats.

### 6.3 Ageing leads to a sleep apnea‐like phenotype in NMS but not controls

In rats, apneas consist of prolonged cessation of respiratory activity (2 breathing cycles; >2 s) that mainly occurs after a sigh (Figure 5A). In young adults, NMS does not affect apnea frequency, and in control females, ageing is associated with a progressive decrease in apneas. As this drop does not occur in NMS females, the apnea frequency measured at old age is higher than that of age‐matched controls (Figure 5B). While the ageing trajectory of NMS females does not accurately reproduce the age‐related rise in SA in women, the progressive divergence in respiratory phenotype between NMS and controls is consistent with the higher SA prevalence in menopausal women. Excessive responsiveness to respiratory stimuli is one of the multiple mechanisms contributing to respiratory instability and apneas during sleep.^93^ NMS‐related increase in the ventilatory response to CO_2_ observed in old females can therefore contribute to the problem, but the fact that a similar effect was not associated with an augmented propensity for apneas in young adults suggests that other mechanisms also contribute.

**FIGURE 5:**
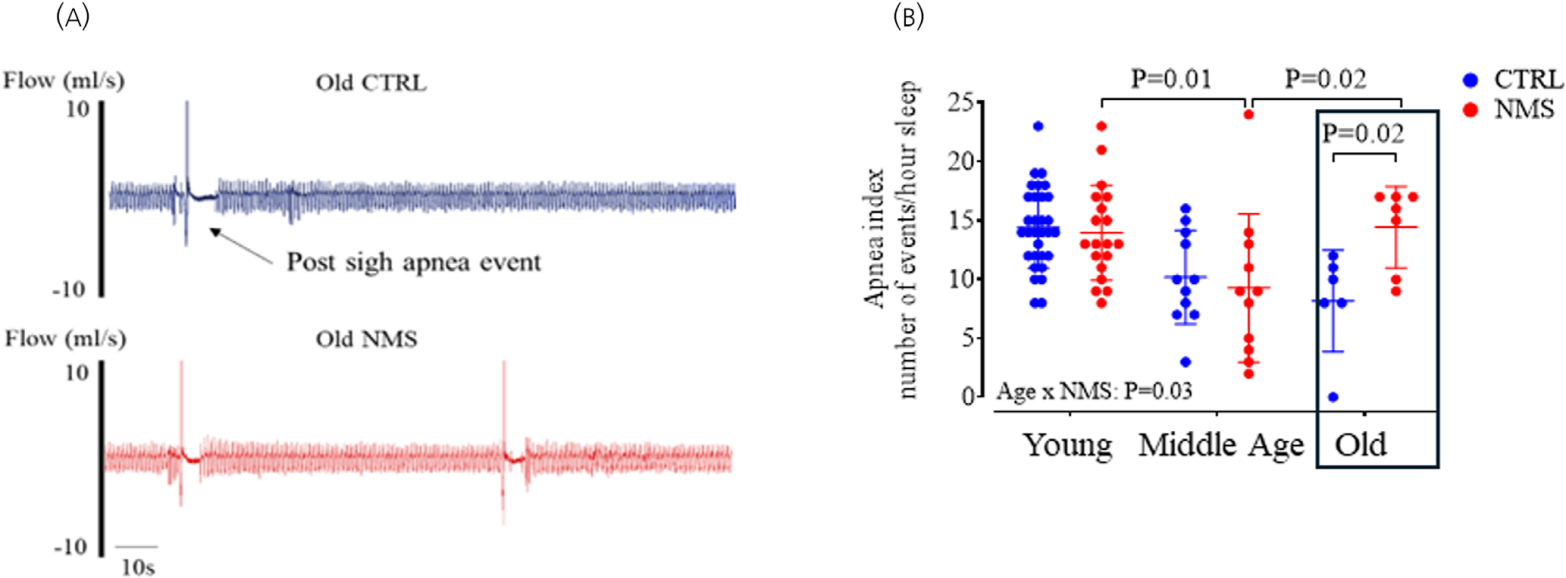
Age‐specific effect of NMS on the apnea index during non‐REM sleep. (A) Original plethysmography recordings comparing respiratory activity during non‐REM sleep from old CTRL (blue) and NMS (red) female rats. (B) Population data comparing the apnea index (total number of apneic events per hour of non‐REM sleep) across age groups and between CTRL and NMS females. Each individual point represents an individual animal breathing with mean ± SD. Modified, with permission, from^102^ under the terms of the Creative Commons Attribution License © 2025.

Elevated body weight is an important risk factor for SA, as it promotes airway collapse, particularly in the supine position. Accordingly, body mass index (BMI) correlates positively with SA prevalence and severity, particularly in younger men.^93^ Body mass index often increases at menopause and while this alone cannot explain the increased risk and severity of SA between pre‐ and post‐menopausal women,^93^, ^94^ BMI exacerbates the impacts of respiratory control anomalies on breathing during sleep.^95^ Comparison of age‐related changes in BMI showed that the augmentation measured in NMS females was greater than in controls. Evaluation of body composition with MRI showed that the proportion of body mass in fat augments progressively with age; however, this gain was greater in NMS than in control (Figure 6A,B). These results indicate that, in ageing females, NMS promotes an obesity‐like phenotype that, when combined with disruption of respiratory control, may augment sleep‐related respiratory disturbances. This interpretation is consistent with clinical data and offers a plausible explanation for the increased prevalence of obesity and SA in a subset of women. Biochemical analyses of plasma samples were then performed to explore potential mechanisms.

**FIGURE 6:**
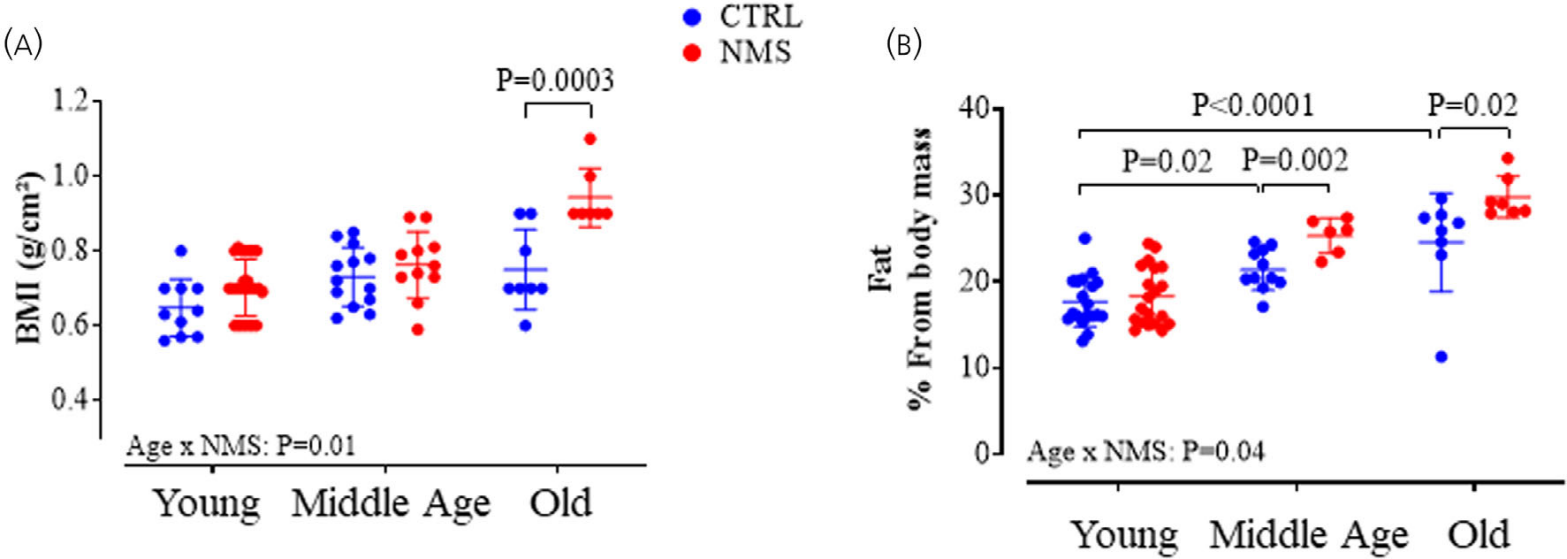
NMS‐related increase in morphology and body composition emerges at old age. Selected indicators of metabolism were measured including: (A) Body mass index, calculated as the ratio of body size over weight. (B) Nuclear magnetic resonance was used to measure percentage of fat. Each individual point represents a value from a CTRL (blue) or NMS rat (red) with mean ± SD. The ANOVA result is reported on the panels. Modified, with permission, from^102^ under the terms of the Creative Commons Attribution License © 2025.

Leptin is an adipocyte‐produced hormone whose main role is to regulate energy balance by inhibiting hunger through hypothalamic networks; however, there is growing preclinical evidence indicating that its actions on respiratory control play a key role in the pathogenesis of SA. Leptin potentiates the hypoxic response and neuromuscular responses to upper airway obstruction,^95^ which contribute to maintaining airway patency. Consistent with the age‐related rise in body fat, leptin levels progressively increase with age; however, NMS did not affect this relationship.^102^

Ageing is naturally associated with augmented circulating levels of inflammatory cytokines,^103^, ^104^ a phenomenon characterised by 2‐ to 4‐fold elevations in cytokines like TNF‐α, IL‐6 and IL‐1β.^104^ Multiple factors contribute to this age‐related immune shift, including increased adipose tissue and decreased sex steroid production. Inflammation contributes to the pathogenesis of SA by reducing airway function due to tissue swelling and impairing neuromuscular control of the airways.^105^ Moreover, inflammation alters ventilatory chemoreflexes, often enhancing hypoxic sensitivity via the carotid body, while producing variable effects on the CO_2_ at the central level.^106^ In our hands, IL‐4, IL‐1β, IL‐6, and IL‐10 levels augmented progressively with age and the highest concentrations detected were in old females. This pattern suggests that ageing was associated with a broader remodelling of immune signalling, involving both pro‐inflammatory mediators, such as IL‐1β and IL‐6, and anti‐inflammatory or regulatory cytokines, such as IL‐4 and IL‐10. Age‐related changes were similar in both groups except for IL‐6 which, at old age, was significantly greater in NMS females than controls.^102^ As IL‐6 primarily has pro‐inflammatory effects, it may contribute to the SA‐related phenotype observed in aged NMS females,^107^ but because our rats were not instrumented to monitor respiratory efforts, we do not know if airway obstruction was the cause of apneic events.

### 6.4 Neonatal maternal separation increases the risk of hypertension in ageing females

In addition to being a major cause of mortality, hypertension is another key co‐morbidity of SA. Much like SA, hypertension has long been considered “a man's disease” owing to its higher prevalence in young males. Yet the prevalence of hypertension in women rises rapidly following menopause and exceeds that of men at about age 60.^98^, ^99^ To determine whether prior exposure to stress affects the cardiovascular ageing trajectory of females, we performed longitudinal monitoring of cardiovascular function in NMS and control rats (Figure 1). In young females, the mean arterial pressure (MAP) of NMS females was 10 mm Hg lower than controls; however, ageing reversed this effect (Figure 7). By comparison with their age‐matched controls, the MAP of NMS females recorded at middle‐ and old‐age were 21‐ and 10‐mm Hg higher (Figure 7). The MAP rise in old NMS females was associated with a 15% decrease in heart rate by comparison with age‐matched controls.

**FIGURE 7:**
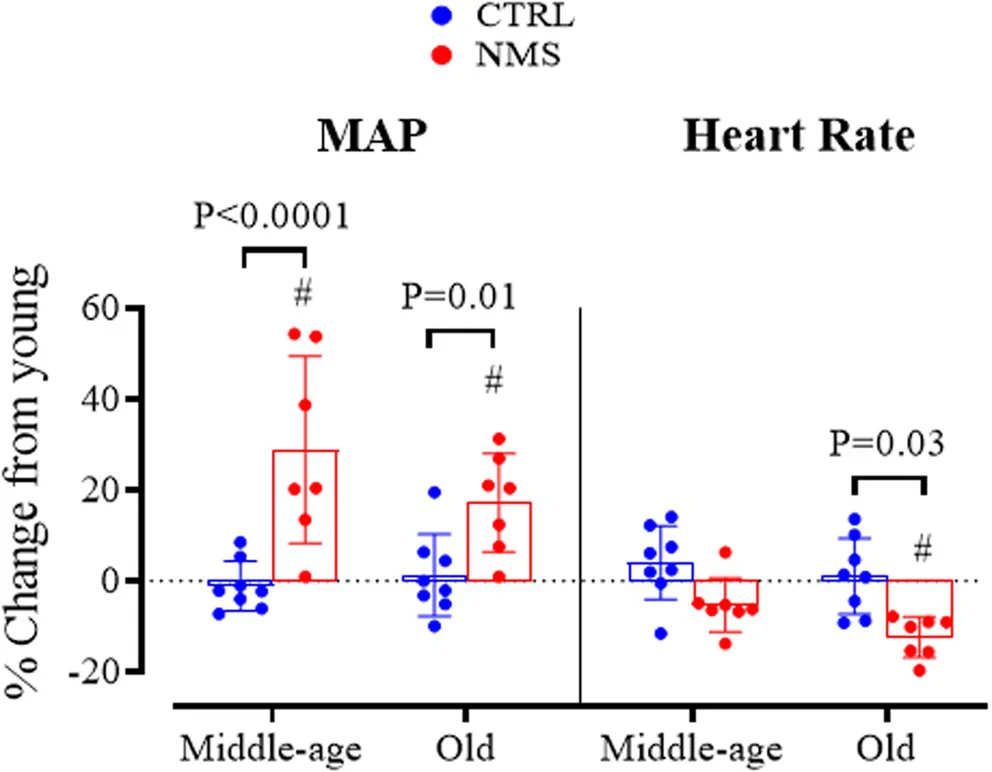
Age‐related changes in mean arterial pressure (MAP) and heart rate in CTRL and NMS females. For each animal, data are expressed as a percentage change from the corresponding mean value measured when the animal was young. Bar height indicates mean value ± SD. ANOVA results are reported under each figure. *p*‐value for the post hoc test is indicated in the figure and significant *p*‐values are highlighted in bold. Modified, with permission, from^24^ under the terms of the Creative Commons Attribution License © 2026.

The renin‐angiotensin system is a multi‐organ hormone pathway that regulates blood pressure and fluid balance. Our interest in this system is based on the fact that sex steroids affect many of its components and thus contribute to many sex‐based differences in blood pressure homeostasis.^99^ Angiotensin converting enzyme (ACE) and its homologue angiotensin converting enzyme II (ACE2) are key elements of this system and play opposing roles in blood pressure homeostasis. By converting angiotensin I to angiotensin II, ACE favours vasoconstriction whereas ACE2 converts angiotensin II to angiotensin 1–7, thus promoting vasodilation. Comparing enzyme activity between NMS and controls using a fluorometric assay showed that NMS did not affect ACE activity at either age; however, ACE2 activity of young NMS rats was 42% higher than in controls.^24^ The pattern observed in old females was inversed, as the ACE2 activity in NMS was now 35% lower than controls. Correlation analysis revealed no relationship between ACE2 activity and MAP in young females; a strong inverse correlation was observed in older animals.^24^ While a reduced capacity for vasodilation is a likely mechanism, it does not exclude the possibility that NMS had other effects contributing to hypertension, including augmented sympathetic activity and/or atherosclerosis. These results reinforce the notion that the deleterious impact of early life stress on the renin–angiotensin system is an important mechanism in the long‐term cardiovascular vulnerability we observed in NMS females.

17*β*‐Estradiol (E2) is another important player in sex‐based differences in cardiovascular function as it improves endothelial function, reduces smooth muscle contractility, sympathetic activity, and MAP in preeclamptic women.^108^ In line with those actions, correlation analysis revealed an inverse relationship between E2 and MAP in NMS females (Figure 8). As this correlation was not observed in controls, these data indicate that NMS augmented E2 sensitivity,^24^ which is consistent with the enhanced action of E2 supplementation on the CO_2_ response of NMS females described in Part I.^32^

**FIGURE 8:**
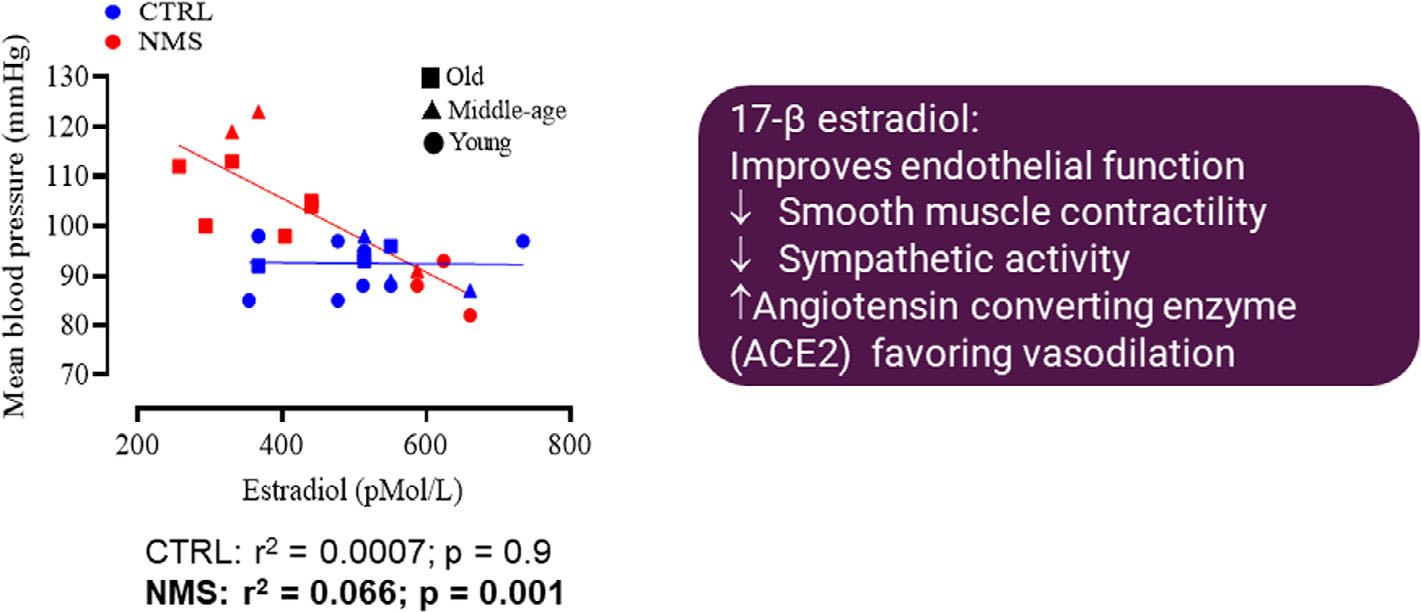
Correlation analyses of the relationships between mean arterial pressure and 17β‐estradiol. Data are reported for females subjected to neonatal maternal separation protocol (NMS, red symbols; 3 h/day from postnatal days 3 to 12) or maintained under standard animal care over the same period (control; CTRL, blue symbols). The origin of the age group of each value is indicated by a distinct symbol: ● young, ▲ middle‐age, and ■ old. Modified, with permission, from^24^ under the terms of the Creative Commons Attribution License © 2026.

### 6.5 Part II: Limitations and conclusion

While our work emphasises E2's contributions to cardiorespiratory disturbances, the potential contributions of progesterone and testosterone to these effects were not explored rigorously, and without comparing how hormone replacement therapy impacts MAP in both groups, it is not possible to draw more definitive mechanistic conclusions. At first, the decline in ovarian hormones alone seems sufficient to reveal the latent effects of early life stress on cardiorespiratory function; however, the impacts of acute (surgically induced) decline in ovarian hormone levels on key neuroendocrine and physiological outcomes suggest otherwise. On the one hand, our recent data showing that in young females, OVX augments FosB labelling in the PVH of NMS but not control females supports E2‐mediated inhibition of the stress pathways (Figure 9A). On the other hand, the lack of NMS‐related increase in stress hormones or FosB labelling in the PVH of old females does not support this (Figure 9B). Furthermore, OVX does not augment MAP or the apnea index of young NMS females (Figure 9C,D). These observations suggest that reproductive ageing and surgical ovarian hormone withdrawal may not engage the same neuroendocrine mechanisms, or may do so with different timing, intensity, or compensatory adaptations. Clearly, further work is required to fully reconcile those observations. As NMS is not acute stress, it is possible that the impact of NMS on the HPA axis declines with age; alternately, the actions of ovarian hormones on the stress pathway relationship differ with ageing. More importantly, these results remind us that while OVX is a useful experimental tool to reduce ovarian hormones, its impacts are not necessarily equivalent to those resulting from progressive reproductive senescence. Natural ageing is associated with multiple changes, including increased adiposity and inflammation, which likely have synergistic effects with ovarian hormone decline. Nevertheless, our results strongly support the concept that experiencing adversity during early life imprints a latent cardiorespiratory vulnerability that is revealed by reproductive senescence.

**FIGURE 9:**
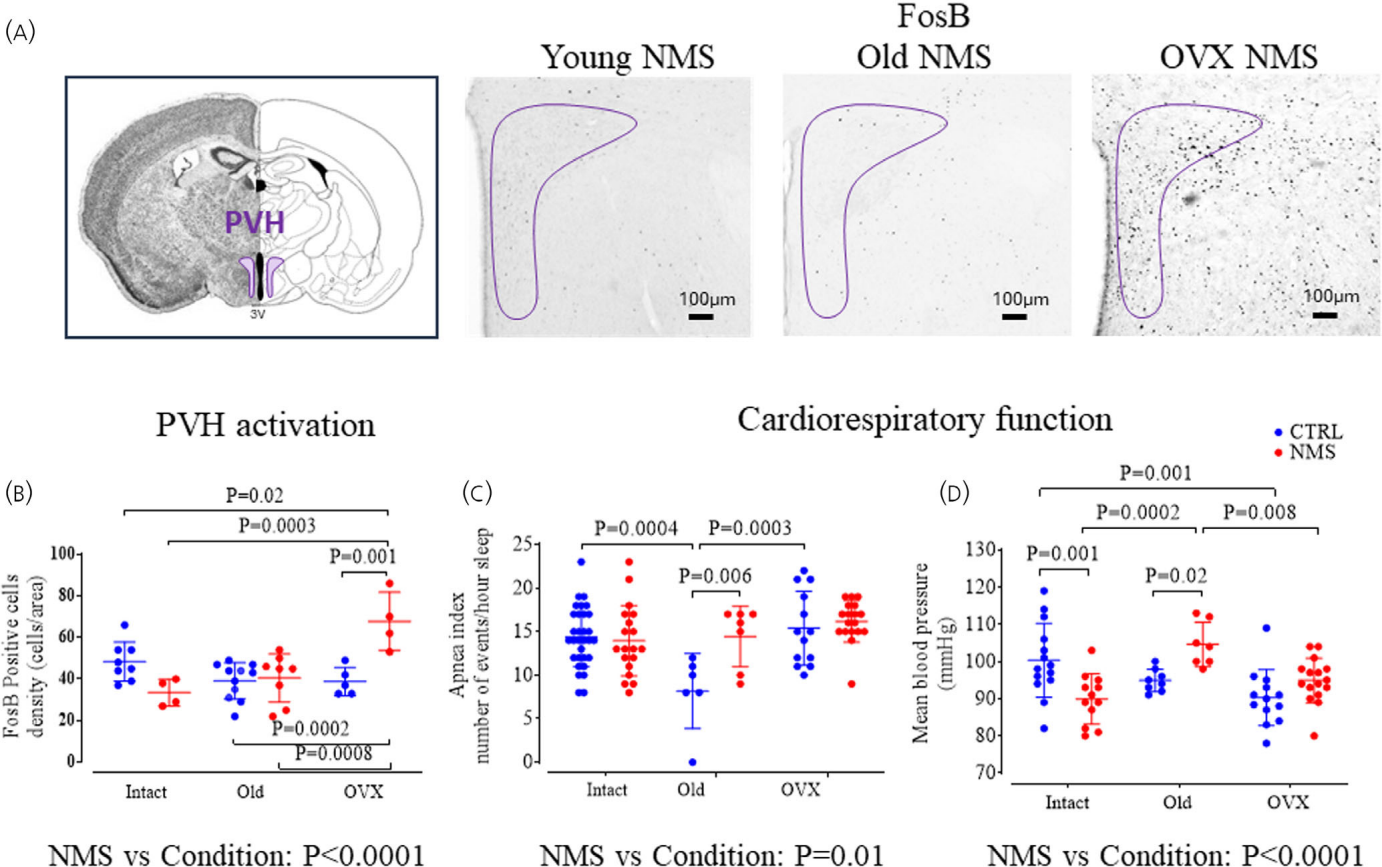
Effect of reproductive ageing and surgical ovarian hormone withdrawal on PVH activation in NMS and control females. (A) Schematic representation of a frontal section indicating the location of the PVH and the area analysed. (B) Photomicrograph comparing FosB observed in NMS of females in three different conditions of ovarian function: Young females with intact ovaries, old females (430–450 days old), and young females 3‐weeks post ovariectomy (OVX). (C) Quantification of FosB perikaryal immunolabelling the PVH in CTRL and NMS females from the different conditions of ovarian function. Data are expressed as cell density. Effects of NMS and ovarian condition on (D) apnea index and (E) mean arterial pressure. Bar height indicates mean value ± SD. Significant *p*‐values of post hoc analyses are highlighted in bold. Modified with permission from^24^ under the terms of the Creative Commons Attribution License © 2026.

## 7 CONCLUSION

The findings reviewed here demonstrate that early‐life stress has neuroendocrine and physiological consequences that differ between sexes which can emerge at various life stages. In females, NMS‐related enhancement of the CO_2_ response, a biomarker of PD, becomes detectable at puberty whereas respiratory instability during sleep, inflammation, increased body fat, and elevated blood pressure develop with ageing. In this framework, ovarian hormones appear to buffer some, but not all, consequences of early adversity. Figure 10 presents a schematic summary of our main results indicating that stress‐related changes in estradiol's actions are an important mechanism. Potential mechanisms (which are not mutually exclusive) include: differences in the relative expression of ERα versus ERβ and changes in aromatase expression. Moreover, NMS‐related changes in these specific components of E2 responses are likely site‐specific. While attractive, those hypotheses require adequate experimental testing.

**FIGURE 10:**
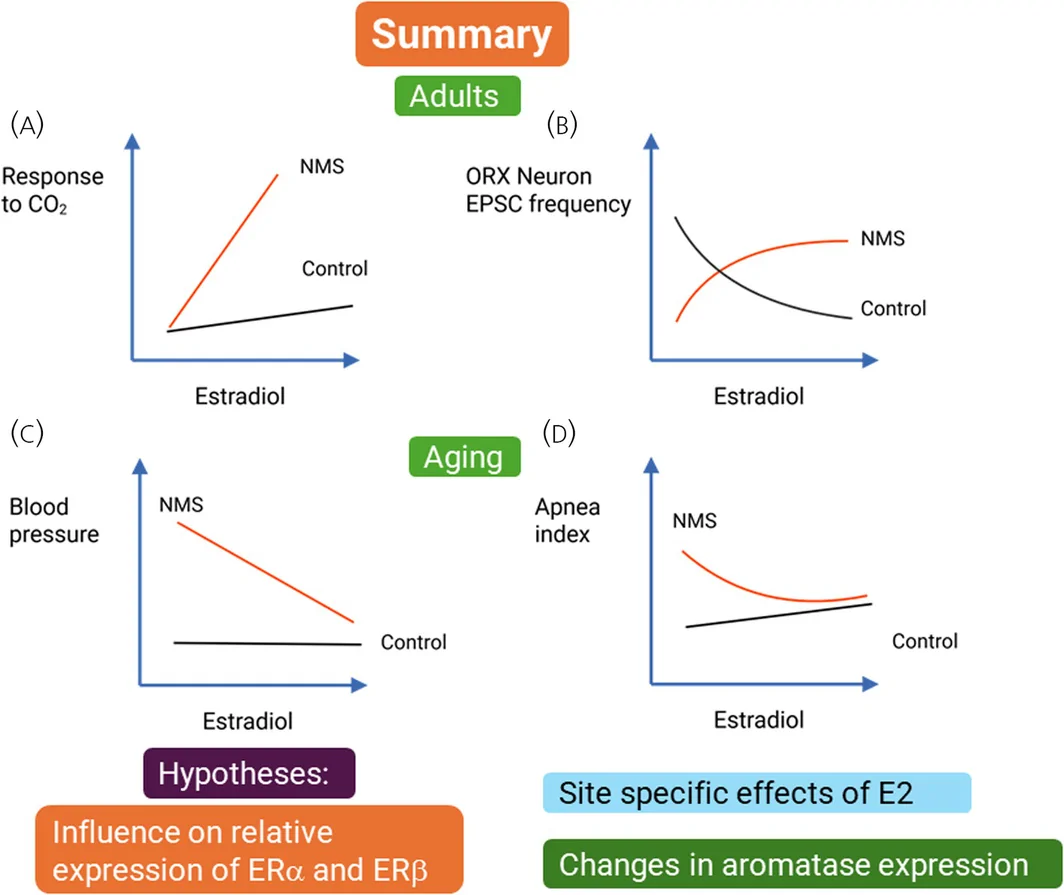
Schematic summary of NMS‐related changes in estradiol's actions. Relationships between E2 and (A) ventilatory response to CO_2_ inhalation (Part I), (B) frequency of excitatory post‐synaptic currents (EPSCs) converging onto orexin‐producing neurons (ORX) (Part I), and (C) mean arterial blood pressure (Part II). Relationships observed for NMS and control rats are reported separately. Created with Biorender.

## AUTHOR CONTRIBUTIONS

**Richard Kinkead:** Conceptualization; funding acquisition; writing – review and editing; writing – original draft; supervision; resources; data curation; project administration. **Stéphanie Fournier:** Writing – review and editing; data curation; supervision; resources; project administration. **Luana Tenorio‐Lopes:** Writing – review and editing; project administration; formal analysis. **Danuzia Ambrozio‐Marques:** Writing – review and editing; project administration; formal analysis.

## FUNDING INFORMATION

This research is supported by: Canadian Institutes of Health Research (CIHR) operating grant PJT‐173396 (Richard Kinkead). Fondation Institut Universitaire de Cardiologie et de Pneumologie de Québec (Richard Kinkead). National Council for Scientific and Technological Development CNPq–Brazil 206239/2014‐9‐PDE (Luana Tenorio‐Lopes).

## CONFLICT OF INTEREST STATEMENT

The authors declare no conflict of interest.

## ETHICS STATEMENT

Details concerning institutional approval for ethical use of research animals are presented in the original studies discussed in this review.

## PERMISSION TO REPRODUCE MATERIAL FROM OTHER SOURCES

The authors have obtained the permissions to reproduce figures from their previous publications in this review.

## Data Availability

The data that support the findings of this study are available from the corresponding author upon reasonable request.
